# Safety in numbers?

**DOI:** 10.1038/s44319-024-00146-1

**Published:** 2024-05-02

**Authors:** Howy Jacobs

**Affiliations:** Tampere University, Tampere, Finland

**Keywords:** Careers, Science Policy & Publishing

## Abstract

How many PhD students per PI is the optimal number?

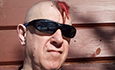

A colleague recently complained to me about how unfair it was that other professors, allegedly with ‘over 20 PhD students already’, were applying for project funding to hire yet more graduate students; whereas other PIs, such as themselves, had only a handful of trainees and very little competitive research funding. The bigger labs, so ran the argument, inevitably appear more productive, attracting ever more resourcing, even if their research output ‘per capita’ is comparatively meagre.

My initial response was to point out that the claim of other PIs having more than 20 students was grossly exaggerated, and that the quality of potential supervision was anyway an almost universal criterion in evaluating funding applications. Nevertheless, my colleague did have a debatable point, in that some PIs do accumulate an excessive number of PhD students, even if the total rarely exceeds 19. It begs the question of what is an appropriate number of disciples to which a PI should aspire. Should this increase, as a PI moves up the academic ladder, and if so by how many? What is the meaning of the student:PI ratio anyway when many research programmes are pursued by large consortia instead of the traditional ‘one (wo)man and a dog’ model? And should we, as a society, try to calibrate the number of PhD students in training to the number of postdoctoral jobs in academia and industry? If so, how?

In the life sciences, the global expansion in research funding from the 1960s through to the present day has fuelled a massive increase in the number of students trained to doctoral level. But this expansion obviously cannot be infinite. Even if only one in five PhD graduates goes on to eventually become a PI in academic science, if each of them generates one new PhD graduate annually over a 40-year career, the number of career scientists will eventually populate the entire globe after a century or two of such exponential progression. A global funding hiatus would sooner or later come into play, so this scenario could never happen. But when the shutters eventually did come down, a large cohort of unemployed and profoundly disillusioned experts would be left wondering what to do with their lives. In fact, the number of PhD graduates already far exceeds the number of jobs in frontline science. The resulting intense competition is a major factor for demotivation, a plethora of mental-health problems and instances of scientific fraud.

Too large a cohort of PhD students is inherently disadvantageous to all members of a lab. It becomes easy for a student experiencing problems to hide away. As a result, those needing the most support are likely to receive the least. It is also difficult for a PI to manage a large group without being perceived, rightly or wrongly, as having ‘favourites’, sowing dissent and discord in the lab, especially when some are succeeding in their project, and thus commanding greater attention, while others may be struggling and in dire need of more guidance and advice. Conversely, it is also a daunting prospect to be the sole or the first PhD student of a PI. Each party is heavily invested in the success of the other, but the result can be an overly intense or even fractious relationship.

Even if we somehow reach an equilibrium where the number of fresh PhD graduates matches the availability of jobs, the question remains as to what is the best model under which to organize their training. In principle, this could be in large graduate schools where a few experts guide many individuals towards making the substantial and original contribution to knowledge that for most of us defines a doctoral award. Or, at the opposite end of the scale, it could be accomplished by more traditional one-on-one mentorship, considered by many a rather archaic model, but which still prevails in many universities.

In both cases, there is a danger of training becoming a kind of indoctrination, where the master’s view of the world is passed on unquestioningly to the next generation: with originality and conceptual advances downgraded or even suppressed. One could argue that the large grad-school model is the best way to avoid this danger, because the number of students vastly exceeds the number of teachers. But dictatorships have long used such a strategy to limit the ability of successive generations of intellectuals to think for themselves. The one-on-one apprenticeship model at least cultivates a diversity of views, but there is still a danger that these views can fossilize from one academic generation to the next.

The prevalent view nowadays seems to come down somewhere in the middle. Essentially a one-on-one ‘parenting system’, tempered by input from a broader thesis committee or adjunct supervisors. The latter are supposed to track the student’s progress, evaluate and nurture their independent thinking, identify personality clashes before they become overtly destructive, and ensure that the supervisor isn’t simply delaying graduation because the student is a source of cheap labour. If properly implemented, I believe that this compromise is a gold standard to which we should aspire. On the flipside, it also expands greatly the scale of academic duties, since every PI has mentorship responsibility for three or four times the number of PhD students nominally assigned to their supervision. Furthermore, I have witnessed too many examples of ‘going through the motions’, where for thesis committee members the exercise becomes one of box-ticking rather than meaningful educational input or scientific interaction.

If we are to stick with anything like the traditional structure of a research group led by a single PI, the only way to ensure high-quality academic training is to apply limits on the number of PhD students each PI can have at one time. In my experience, to do the job of graduate supervision properly requires minimally 10% of a PI’s work time per student over the full 4–5 years of a studentship. Being a second supervisor or thesis committee member should take up at least one-third as much effort. Thus, in order to leave any space at all for other academic duties, a PI should have at most five PhD students at one time, even if postdocs and other associates carry some of the burden. It should be up to funders, deans, chairs and directors to make sure this principle is respected: preferably by consent and advice rather than by rigid rules. Every aspiring young scientist should be able to command a substantial slice of their supervisor(s)’ time and intellectual engagement. And the success of a PI should not be measured by the number of PhD theses guided, but by the quality of the guidance and by how well each student’s future career is shaped according to their personal strengths.

In ‘olden times’ it all seemed so much easier. A doctoral degree was the passport to a fulfilling career in science. And there was no pressure to train dozens of others to follow in our footsteps. In the end my colleague has won the argument.

### Supplementary information

Peer Review File

