# Supplementary material for: Safety in numbers?
Source: EMBO Rep. 2024 May 2;25(6):2513–4. doi: 10.1038/s44319-024-00146-1 (PMC11169480; doi:10.1038/s44319-024-00146-1)
Supplement: Supplementary file 1 — Peer Review File [file 44319_2024_146_MOESM1_ESM.pdf]

EMBO reports

Safety in numbers?

Howard Jacobs

Corresponding author(s): Howard Jacobs (howard.jacobs@tuni.fi)

**Review Timeline:**	Submission Date:	12th Apr 24
	Accepted:	17th Apr 24

Editor: Holger Breithaupt

Transaction Report: This article type is not peer reviewed.
